# Meaning Reflectivity in Later Life: The Relationship Between Reflecting on Meaning in Life, Presence and Search for Meaning, and Depressive Symptoms in Older Adults Over the Age of 75

**DOI:** 10.3389/fpsyg.2021.726150

**Published:** 2021-10-28

**Authors:** Laura Dewitte, Jessie Dezutter

**Affiliations:** Faculty of Psychology and Educational Sciences, KU Leuven, Leuven, Belgium

**Keywords:** meaning in life, meaningfulness, old age, ageing, psychological well-being, eudaimonic well-being, depression

## Abstract

**Background:** Over the last decades, there is growing attention for the importance of meaning in life for older adults. However, there is virtually no insight into the mental processes that contribute to this experience. Some scholars recently called for an investigation of meaning reflectivity, or the process of reflecting on issues specifically related to meaning in life. In this study, we explored to what extent older adults talk and think about issues of meaning in life, and how this meaning reflectivity is related to the search for and presence of meaning in life, and to depressive symptoms.

**Method:** In this cross-sectional observational study, 282 community-residing older adults (75 or older) in Belgium filled in paper questionnaires on meaning in life (presence and search), depressive symptoms, and meaning reflectivity (categorical item). ANOVA analyses were used to explore differences in meaning in life and depressive symptoms across the meaning reflectivity categories. Regression and negative binomial models investigated the association between meaning reflectivity and presence, search and depressive symptoms. Finally, an exploratory structural equation model examined whether presence of meaning statistically mediated the relationship between meaning reflectivity and depressive symptoms.

**Results:** The majority of participants (42.4%) indicated that they had thought about meaning in life before, 23.2% indicated that they had talked about it before, 18% indicated that they hadn’t thought about it before but found it interesting, and 16.4% indicated that they were indifferent/unconcerned about meaning in life. The latter group reported lower levels of presence of meaning and search for meaning and higher levels of depressive symptoms. Belonging to this category was also associated with lower presence and search in regression analyses, but not with depressive symptoms above the effect of presence of meaning. Exploratory mediation analyses suggested that presence of meaning may be a mediator between meaning reflectivity and depressive symptoms.

**Conclusion:** Meaning reflectivity is an important process to consider in the context of the experience of meaning in life for older adults. Those older adults who are indifferent about issues of meaning in life might be more vulnerable to experience a lack of meaning and depressive symptoms.

## Introduction

### Meaning in Life in Old Age

More and more, scholars in the field of aging research are pointing to the psychological experience of meaning in life as a crucial variable to consider in the context of positive late life functioning. In contemporary psychology, meaning in life is defined as an interplay of three *components*: (a) purpose in life, or a sense of being directed by valued life aims, (b) significance, or a sense that life matters and is worth living, and (c) coherence, or a sense that life is logical and comprehensible (Heintzelman and King, 2014; George and Park, 2016; Martela and Steger, 2016). Furthermore, the *presence* of meaning in life can be distinguished from the *search* for meaning in life (Steger et al., 2006), in which the former refers to the extent that people tend to experience their lives as purposeful, significant, and coherent, while the later refers to the extent that people tend to make efforts to establish or strengthen this experience (Steger et al., 2008a,b). A sense of meaning in life can be derived from different *sources* of meaning, like family, friends, work, health, religion or spirituality, personal development, leisure activities, creativity, material possessions, etc. (e.g., Debats, 1999; Schnell, 2009; Delle Fave et al., 2013). Both theoretical and empirical work suggest that in the later stages of life, more individualistic sources often tend to make way for more communal and transcendental sources such as spirituality and religion, nature, and community engagement and societal commitment (Bar-Tur et al., 2001; Fegg et al., 2007; Wong, 2016).

Despite the many challenges involved in growing older, many older adults seem able to sustain a solid sense of meaning in life. They may even experience higher presence of meaning than middle aged adults; some research suggests a curvilinear relationship between meaning in life and age, with presence of meaning being higher in early and later life, and search for meaning being lower in early and later life (Fegg et al., 2007; Steger et al., 2009). In other words, from middle adulthood onward, presence of meaning in life tends to increase with age, while search for meaning tends to decrease. On the other hand, a recent study focusing specifically on older adults found that in advanced age, meaning in life may decrease again for the oldest old (85 +) compared to the younger old (Steptoe and Fancourt, 2019). Other findings also suggest that especially the component of purpose in life is likely to decrease somewhat in old age, although there are important inter-individual differences (Pinquart, 2002; Hill and Weston, 2019).

Whether or not an older adult is able to maintain a sense of meaning in life is not a trivial matter. An increasing body of empirical evidence shows that higher levels of presence of meaning or purpose in life in older adults are predictive of a broad range of physical and mental health outcomes, both cross-sectionally and over time (e.g., Krause, 2007; Boyle et al., 2010; Windsor et al., 2015; Irving et al., 2017; Steptoe and Fancourt, 2019; Volkert et al., 2019). This is leading researchers in aging to believe that the presence of meaning in life is an especially important aspect of positive aging. When it comes to search for meaning and its relation to well-being, the picture is less straight-forward. One study found that searching for meaning was more strongly associated with psychological distress and diminished well-being in older adults (Steger et al., 2009). The authors suggested that explorative processes such as searching for meaning may be more developmentally adaptive in earlier life stages such as adolescence. However, in line with findings in the general population (Park et al., 2010), other work suggests that searching for meaning in older adults is mainly related to more psychological distress when these adults also experience low presence of meaning (Van der Heyden et al., 2015).

### The Role of Meaning Reflectivity

Taken together, researchers have made good progress in understanding what the components, sources, and benefits of experiencing meaning in life are for older adults (Reker and Wong, 2012; Irving et al., 2017; Hupkens et al., 2018). However, another important aspect for understanding meaning has received relatively less attention: how does an individual derive a subjective sense of meaning from their sources of meaning? Or in other words, what are the mental processes through which this sense of meaning is arrived at? An exception for the lack of attention for meaning processes comes from the *meaning making* literature. However, this work has focused mainly on meaning making in the face of threats to meaning, like stressful or traumatic events (Heine et al., 2006; Park, 2010). Park’s (2010) meaning making model, for instance, distinguishes between global and situational meaning. The former represents a personal system of overarching life goals and global beliefs about the world and the self; the latter represents the meaning that is encountered in specific situations. In stressful or traumatic events, the situational meaning encountered can violate the expectations from the global meaning system. In response, people may employ a range of different meaning making processes (e.g., positive reappraisal, accommodation of prior beliefs) in an attempt to solve the discrepancy and alleviate distress. This model provides a helpful framework for understanding how people can restore a sense of meaning in challenging circumstances. However, the model provides no account of how a subjective sense of meaning is derived from the global meaning system and sustained on a daily basis (Park and George, 2018).

Although there is almost no insight into the cognitive processes that contribute to the construction and maintenance of a sense of meaning in old age, a limited numbers of studies in the general adult population can provide some direction. Recently, Hill et al. (2019) suggested that an important aspect of daily meaning construction could be *meaning reflectivity*, or the process of reflecting on issues specifically related to meaning in life. They even propose that reflectivity about meaning in life should be added to the conceptualization of meaning in life because “a propensity to reflect on MIL [meaning in life] is as important as concluding that one has it.” (p. 207–208). It is important to note that meaning reflectivity is different from the search for meaning. While the latter is characterized by a desire to augment your sense of meaning, meaning reflectivity does not necessarily originate from a wish to change current circumstances and can appear in individuals who are not searching for more meaning in life. Furthermore, while searching for meaning is focused on meaning in the future, meaning reflectivity can deal with the past, present, and future (Hill et al., 2019). However, as individuals who are searching for meaning likely also reflect on it more, search and reflectivity can be expected to be positively related. Furthermore, if reflecting on meaning is a process that orients people toward finding or constructing meaning in their lives, it can also be expected to be positively related to presence of meaning. In a cross-sectional investigation of their newly developed meaning reflectivity subscale, Hill et al. (2019) indeed found a positive correlation between meaning reflectivity and both presence and search for meaning in a sample of middle-aged US adults. Furthermore, meaning reflectivity also showed a positive correlation with subjective well-being and a small negative correlation with depression and emotional instability. These findings suggest that meaning reflectivity could indeed be an important process through which people derive a sense of meaning in life and which contributes to their well-being.

A few years back, Allan and Shearer (2012) already investigated a similar idea. They developed the scale for “existential thinking” to assess individual differences in “the tendency to engage with ultimate concerns and the capacity to carry out a meaning-making process that locates oneself in relation to these existential issues” (p. 22). This concept is related to the concept of meaning reflectivity but broader, as it also includes other existential themes (e.g., death, the universe, eternity). They hypothesized that this process of existential thinking may be “a critical part in understanding how people establish, discover, or maintain a sense of meaning in their lives.” As preliminary support, they showed that existential thinking was positively related to presence and search for meaning in life and to existential well-being in a broad adult sample, and that meaning in life statistically mediated the relation between existential thinking and existential well-being. However, another study in a similar broad adult population (ages 17–78) only found a positive relation between existential thinking and search for meaning but not presence for meaning (Kretschmer and Storm, 2018).

More indirectly relevant, some studies have focused on the role of a general reflective cognitive style (i.e., the tendency to be self-reflective in general, not specifically focused on meaning or existential issues) for the experience of meaning in life. Being able to think reflectively has indeed been proposed to be a crucial condition for experiencing meaning (Martela and Steger, 2016), but findings are mixed. One study found that reflection was positively related to presence of meaning and positive affect (Boyraz and Efstathiou, 2011) while another found that reflection was predictive of search for meaning and negative affect (Newman and Nezlek, 2019).

Reflecting on meaning in life may also not be unambiguously beneficial for psychological functioning. In a large multi-national study, Joshanloo and Weijers (2014) used a single item to explore whether the extent that people think about the meaning or purpose of life was related to their overall life satisfaction. They found that this relationship was moderated by religiosity and globalization. More specifically, for those high on religious attendance, thinking more about meaning in life was related to higher life satisfaction. For those low in religious attendance, the relation was negative. Moreover, the relation was also more negative for individuals living in more globalized countries. The authors suggest that thinking about meaning in life can be detrimental for well-being when the cultural and societal context does not provide straightforward positive answers to these questions, such as in more secular and globalized societies.

In sum, studies suggest that meaning reflectivity may be a process spurring both the search and the construction of a sense of meaning in life. Through its relation with meaning in life, meaning reflectivity may also be related to outcomes of positive psychological functioning. However, the limited empirical evidence available shows a somewhat mixed picture and no studies have focused on meaning reflectivity specifically in older adults. For older adults, meaning reflectivity could potentially become more prominent. Tornstam’s gerotranscendence theory, for example, proposes that old age is accompanied by “a shift in meta-perspective from a materialistic and rational view to a more cosmic and transcendent one” (Tornstam, 1989, 1997). According to this view, older adults who are nearing the end of life are invited to come to terms with their past lives and prepare for death, which requires a fundamental shift in perspective “from mundane issues to a concern with universal values” (Yount, 2008, p. 81). Importantly, this process involves both reflecting back and looking forward to the future, beyond the self. Other developmental views on aging have similarly suggested that older adults engage in more contemplation and reflection on their lives and its place in the larger scheme of things (Cohen, 2005; Hupkens et al., 2018). Therefore, as life enters its later phases, reflecting on issues of meaning in life may be a core process in the psychological functioning of older adults.

### Present Study

The present study had as objective to explore the frequency and importance of meaning reflectivity in older adults living in the community. More specifically, we examined to what extent older adults aged 75 or older (i.e., the old-old and oldest old) talk and think about issues of meaning in life, and how this meaning reflectivity is related to the search for and presence of meaning in life and to depressive symptoms. We focused on depressive symptoms as a key indicator of late life psychological functioning. Population based studies across different countries suggest that depressive symptoms are prevalent in older adults and increase with age (e.g., Minicuci et al., 2002; van’t Veer-Tazelaar et al., 2008; Glaesmer et al., 2011). Moreover, it is one of the most recurring findings in the empirical meaning literature that older adults with higher levels of meaning in life also have less depressive symptoms. This is in line with the theoretical view of meaning as a human strength and resource for dealing with life challenges (e.g., Frankl, 1968; Davis et al., 1998). We therefore deemed it particularly interesting to examine whether meaning reflectivity could statistically predict additional variation in depressive symptoms above this already established association with presence of meaning in life.

Based on developmental views of aging as a life stage of more deepened contemplation, we expected the majority of older adults to engage in some form of meaning reflectivity (thinking or talking about meaning in life). Based on the limited empirical findings in general adult samples, we also expected that older adults who engaged in thoughts or conversations about meaning in life would have higher rates of presence and search for meaning in life than older adults who did not practice any meaning reflectivity. Furthermore, we hypothesized that these adults would report less depressive symptoms. Previous work suggests that meaning reflectivity may be related to better psychological functioning (Hill et al., 2019). This may be especially so for more religious individuals (Joshanloo and Weijers, 2014). While the present study unfortunately did not include a measure for religion, in secularized Western nations such as the country in which this study was conducted (Belgium), older adults tend to be more religious than younger generations, likely both due to cohort differences and developmental changes (Bengtson et al., 2015).

In addition, we examined whether differences in meaning reflectivity would statistically predict presence of meaning and search for meaning after adjusting for covariates, and would predict depressive symptoms above any predictive effect of presence of meaning, search for meaning, and covariates. As a more exploratory investigation, finally, we wanted to examine the potential mediating role of meaning in life in the relation between meaning reflectivity and depressive symptoms (cf. Allan and Shearer, 2012).

Gaining insight into how much older adults reflect on meaning in life and how this is related to their psychological functioning and overall sense of meaning in life can provide important information for understanding why some older adults struggle with feelings of meaninglessness while others do not, and can help to identify pathways through which these struggles can be tackled.

## Materials and Methods

### Participants and Procedure

Data collection was part of a larger study conducted in Belgium (the TiMe project)^1^, which aimed to investigate older adults’ meaning in life experience and future time perspective. At the end of 2017 and beginning of 2018, Dutch-speaking older adults of 75 years or older living at home (i.e., not in a residential care setting) without an acute medical condition or severe cognitive problems were sampled by three master thesis students through different channels (personal network, senior organizations). Three hundred and seventy six paper questionnaires with informed consent were distributed, of which 325 were completed independently by older adults in their home environment and returned to the researchers. Thirty one of these questionnaires were excluded because they did not contain the completed informed consent. Another 12 participants were excluded because they were younger than 75, leading to a final convenience sample of 282 older adults. Their mean age was 81.9 years (*SD* = 4.33, range 75–94) and 175 of them (62%) were women. Most participants were married (139; 49%), 105 were widowed (37%), 12 living together with a partner (4%), 11 single (4%), 8 divorced (3%), and for 7 participants data on civil state was missing (3%). Most participants had higher secondary education as highest degree (110; 39%), 74 had higher education (college or university; 26%), 61 had lower secondary education (22%), 31 had primary education only (11%), and for 6 participants data on education was missing (2%).

### Measures

#### Meaning in Life (Presence and Search)

The Presence of Meaning and Search for Meaning subscales of the Meaning in Life Questionnaire Short Form were used to assess meaning in life (Steger and Samman, 2012). Each subscale consists of three items which were rated on a scale from 1 (totally disagree) to 4 (totally agree). The original 7-point Likert scale was adjusted to diminish cognitive burden on older participants. An example item for the Presence subscale is: *“I have a clear sense of what makes my life meaningful.”* An example item for the Search subscale is: *“I am seeking a purpose or mission for my life.”* Mean scores were calculated (potential range 1–4) with higher scores indicating higher Presence and Search. In the current sample Cronbach’s alpha was 0.80 for Presence of Meaning and 0.85 for Search for meaning.

#### Depressive Symptoms

An 8-item short version of the Geriatric Depression Scale was used to assess depressive symptoms (Jongenelis et al., 2007). Eight questions (e.g., *“Do you feel that your situation is hopeless?”*) were answered with yes (1) or no (0) by participants. A sum score was calculated (potential range 0–8), with higher scores indicating higher burden of depressive symptoms. Cronbach’s alpha in this sample was 0.71.

#### Meaning Reflectivity

One item with four answer categories was included at the end of the questionnaire to assess meaning reflectivity: *“Before completing this questionnaire, had you ever thought about meaning in your life before?”* The four answer categories were: (1) Yes, I have talked about this with others, (2) Yes, I have thought about it before, (3) No, but I do find it interesting, (4) No, that doesn’t concern me.

#### Demographic Variables

Participants reported on age, gender, civil status and level of education (from 1 “primary education” to 4 “higher education”). Civil status was recoded into two broad categories: (1) married or living together, (2) single, divorced or widowed.

### Data Analyses

Statistical analyses were performed in SPSS and R. We first explored missing data and calculated descriptive statistics, Pearson correlations between continuous variables, and differences in the main variables across different demographic categories (gender, civil status). The primary analyses existed of different steps. First, ANOVA analyses with *post hoc* Tukey tests were used to examine whether participants in the different meaning reflectivity categories scored differently on presence of meaning, search for meaning, and depressive symptoms.

Second, regression analyses were used to examine the association between meaning reflectivity and presence and search for meaning. In addition, the unique predictive effect of meaning reflectivity, presence of meaning and search for meaning for depressive symptoms was examined. Because the outcome variable of this model (Geriatric Depression Scale) is count data, a negative binomial model was used. This model was preferred over a Poisson regression because there was overdispersion of the outcome variable (i.e., the variance was larger than the mean) (Gardner et al., 1995). The regression and negative binomial models were analyzed in SPSS. Age, gender, education and civil status were included as covariates and multiple imputations were used to handle missing data.

Third, in case the previous models suggested a significant relation between meaning reflectivity, meaning in life, and depressive symptoms, we planned to examine whether meaning in life statistically mediated the relationship between meaning reflectivity and depressive symptoms. Given the cross-sectional data, this analysis was regarded as an exploratory indication for future studies and was interpreted with caution. The mediation model was tested in R using the lavaan package (Rosseel, 2012) for structural equation modeling. Because this package does not provide an option for count outcomes, we used the MLR estimator which is robust against non-normality. Full Information Maximum Likelihood (FIML) was used to handle missing data.

## Results

### Preliminary Analyses

Descriptive statistics including rates of missing data and Pearson correlations are displayed in Table 1. Meaning reflectivity showed the largest percentage of missing data (11.3%). Participants who did not fill in this question were significantly older (*M* = 84.74, *SD* = 3.95) than participants who did (*M* = 81.57, *SD* = 4.25), *t*(259) = 3.70, *p* < 0.001, but did not differ significantly on presence of meaning, search for meaning or depressive symptoms. Crosstabs with Chi-Square tests suggested that rate of missingness on meaning reflectivity did not significantly differ across categories of gender, civil status or education (full missing data analyses output on the OSF project page).^2^ Subsequent analyses were performed under the assumption of data missing at random (MAR) (Graham, 2009).

**TABLE 1:** Missing data, means, standard deviations, and correlations with confidence intervals.

Variable	*n* (% missing)	Potential range	Observed range	*M*	*SD*	1	2	3	4
1. Age	261 (7.4%)			81.90	4.33				
2. Education	276 (2.1%)			2.82	0.95	–0.14*			
						[–0.26, –0.02]			
3. Presence of Meaning	264 (6.4%)	1–4	1–4	3.02	0.70	–0.02	0.14*		
						[–0.14, 0.11]	[0.02, 0.26]		
4. Search for Meaning	258 (8.5%)	1–4	1–4	2.20	0.80	–0.09 [–0.21, 0.04]	0.19** [0.07, 0.31]	0.36** [0.25, 0.46]	
5. Geriatric Depression Scale	278 (1.4%)	0–8	0–6	0.40	0.97	0.13* [0.01, 0.25]	–0.23** [–0.34, –0.11]	–0.28** [–0.38, –0.16]	–0.14* [–0.26, –0.02]
6. Meaning reflectivity	250 (11.3%)								

M and SD are used to represent mean and standard deviation, respectively. Values in square brackets indicate the 95% confidence interval for each correlation. The mean education reflects an education level between lower and higher secondary education. * indicates p < 0.05. ** indicates p < 0.01.

With regard to the relation between demographic variables and the main variables, age showed a small positive correlation with depressive symptoms. Level of education showed a small positive correlation with both presence of meaning and search for meaning and a small to moderate negative correlation with depressive symptoms (Table 1). Independent *t*-tests showed that there were no significant gender differences in presence of meaning, search for meaning, or depressive symptoms. Regarding civil status, participants who were married or living together scored significantly higher on search for meaning (*M* = 2.28, *SD* = 0.80) than participants who were widowed/single/divorced (*M* = 2.06, *SD* = 0.79), *t*(249) = 2.20, *p* = 0.029.

### Comparison of the Meaning Reflectivity Categories

In response to the question of whether participants had considered the topic of meaning in life before completing the questionnaire, 106 of 250 participants (42.4%) who provided an answer to this question indicated that they had *thought* about meaning in their life before the study (“Yes, I have thought about it before”). Another 58 participants (23.2%) indicated that they had *talked* about it before (“Yes, I have talked about this with others”). Forty-five participants (18%) indicated “No, but I do find it interesting,” while 41 participants (16.4%) indicated that they were not interested in the topic (“No, that doesn’t concern me.”).

ANOVA analyses revealed that participants in different meaning reflectivity categories had significantly different scores on presence of meaning, search for meaning, and depressive symptoms (see Table 2). Participants who indicated that they were unconcerned with meaning in their life (Category 4) reported significantly lower presence of meaning and search for meaning in their lives than participants in the other three categories. They also reported more depressive symptoms than participants who had thought about meaning before (Category 2) or found it interesting (Category 3). Participants who had talked or thought about meaning (Categories 1 and 2) scored significantly higher on search for meaning than participants who hadn’t thought about meaning in their lives before the study (Categories 3 and 4).

**TABLE 2:** Mean values of the main outcome variables for the four meaning reflectivity categories with univariate ANOVA tests and post hoc comparisons.

Outcome variable (potential range)	Meaning reflectivity						*n*	η ^2^
	Category 1 (talked about)	Category 2 (thought about)	Category 3 (interesting)	Category 4 (unconcerned)	*F*-value (*df*)		
Presence of Meaning (1–4)	3.07 (0.65)^a^	3.16 (0.60)^a^	3.07 (0.67)^a^	2.40 (0.81)^b^	12.93 (3, 239)***	243	0.14
Search for Meaning (1–4)	2.56 (0.77)^a^	2.38 (0.75)^a^	2.01 (0.72)^b^	1.44 (0.45)^c^	22.99 (3, 230)***	234	0.23
Geriatric Depression Scale (0–8)	0.41 (0.88)	0.18 (0.54)^a^	0.30 (0.70)^a^	0.85 (1.44)^b^	6.19 (3, 243)***	247	0.07

Standard deviations in parentheses. Post hoc comparisons using Tukey HSD tests. Mean values with different superscripts (a, b, c) are significantly different from each other. Means without any superscript do not differ significantly from any other mean. Category 1 = “Yes, I have talked about this with others,” Category 2 = “Yes, I have thought about it before,” Category 3 = “No, but I do find it interesting,” Category 4 = “No, that doesn’t concern me.” ***p < 0.001.

### Meaning Reflectivity as Statistical Predictor of Meaning in Life and Depressive Symptoms

Table 3 shows the results of the regression models and the negative binomial model. The table includes pooled estimates based on 20 imputed datasets (Graham, 2009). Presence of meaning, search for meaning, depressive symptoms, and meaning reflectivity were imputed at the scale-level using an imputation model which included all the variables from the regression and negative binomial model and additional auxiliary variables with possible predictive value not used in the current study (Sterne et al., 2009; full imputation syntax on OSF (see text footnote 1).

**TABLE 3:** Summary of regression models for predicting presence of meaning and search for meaning (left columns) and the negative binomial model predicting the geriatric depression scale (right column).

	Presence of Meaning			Search for Meaning			Geriatric Depression Scale		
	*B* [95% CI]	*SE*	*p*	*B* [95% CI]	*SE*	*p*	*B* [95% CI]	*SE*	*p*
Intercept	1.93* [0.11, 3.75]	0.92	0.04	2.87** [0.79, 4.96]	1.06	0.007	–3.60 [–8.59, 1.40]	2.55	0.16
Age	0.002 [–0.02, 0.02]	0.01	0.85	–0.02 [–0.04, 0.01]	0.01	0.17	0.07* **[0.01, 0.13]**	0.03	0.02
Education	0.09 [–0.01, 0.19]	0.05	0.08	0.10 [–0.02, 0.21]	0.06	0.09	–0.42** **[–0.72, –0.11]**	0.16	0.008
Gender									
Men	0.16 [–0.04, 0.36]	0.10	0.12	–0.02 [–0.25, 0.21]	0.12	0.88	0.15 [–0.46, 0.76]	0.31	0.63
Women^*a*^	–	−	−	–	−	−	–	−	−
Civil status									
Married/living together	0.11 [–0.08, 0.30]	0.10	0.27	0.12 [–0.10, 0.34]	0.11	0.28	–0.50 [–1.12, 0.13]	0.32	0.12
Single/divorced/widowed^*a*^	–	−	−	–	−	−	–	−	−
Meaning Reflectivity									
Category 1 (talked about)	0.33* **[0.06, 0.61]**	0.14	0.02	0.60** **[0.20, 1.00]**	0.20	0.004	–0.18 [–1.23, 0.87]	0.53	0.74
Category 2 (thought about)	0.48** **[0.20, 0.76]**	0.14	0.001	0.51* **[0.12, 0.89]**	0.19	0.01	–0.65 [–1.67, 0.37]	0.52	0.21
Category 3 (interesting)	0.43* **[0.10, 0.76]**	0.17	0.01	0.16 [–0.29, 0.60]	0.22	0.49	–0.36 [–1.46, 0.73]	0.55	0.51
Category 4^*a*^ (unconcerned)	–	−	−	–	−	−	–	−	−
Presence of Meaning							–0.68** **[–1.11, –0.24]**	0.22	0.002
Search for Meaning							–0.01 [–0.43, 0.42]	0.22	0.98

N = 249 (complete cases after multiple imputations). Pooled estimates of 20 imputed data sets to handle missing data on presence of meaning, search for meaning, and meaning reflectivity. B, unstandardized coefficients; SE, standard errors; CI, confidence interval. Significant predictor coefficients in bold. Category 1 = “Yes, I have talked about this with others,” Category 2 = “Yes, I have thought about it before,” Category 3 = “No, but I do find it interesting,” Category 4 = “No, that doesn’t concern me.” ^*a*^Reference category. **p < 0.01; *p < 0.05.

The two left columns show the regression models predicting presence of meaning and search for meaning. Compared to the reference category (unconcerned with meaning in life), belonging to any of the other three categories of meaning reflectivity was significantly associated with higher scores on presence of meaning in life. For search for meaning, belonging to one of the first two categories (talked or thought about meaning in life before the study) was significantly associated with higher scores on search for meaning compared to the reference category.

The right column of Table 3 shows the results of the negative binomial model predicting depressive symptoms. Higher age, lower level of education, and lower presence of meaning was significantly associated with a higher level of depressive symptoms. Meaning reflectivity was not significantly associated with any additional variance in depressive symptoms.

### Meaning in Life as Mediator Between Meaning Reflectivity and Depressive Symptoms

The regression and negative binomial models indicated that meaning reflectivity was a significant statistical predictor of presence of meaning and search for meaning, and that presence of meaning (but not search for meaning) was a significant predictor of depressive symptoms. We therefore tested an exploratory mediation model (using a structural equation model framework) to examine whether presence of meaning was a statistical mediator between meaning reflectivity and depressive symptoms. The mediation model had excellent fit [χ^2^(1) = 1.39, *p* = 0.24; CFI = 0.996; TLI = 0.938; RMSEA = 0.033; SRMR = 0.007]. A summary of the model output is shown in Table 4. The model suggests a partial mediation for category 2 (thought about meaning in life before) compared to the reference category (unconcerned with meaning in life) in the prediction of depressive symptoms, with both a significant direct and indirect effect. For category 1 and 3 (talked about meaning and interested in meaning), only the indirect was significant, suggesting a full mediation through presence of meaning in predicting depressive symptoms.

**TABLE 4:** Summary of mediation model with presence of meaning as mediator between meaning reflectivity and depressive symptoms.

Outcome	Predictor		*B* [95% CI]	β	*SE*	*P*
Presence of Meaning	Meaning reflectivity					
	Category 1 (talked about)	a1	0.62 [0.33, 0.91]	0.37***	0.15	< 0.001
	Category 2 (thought about)	a2	0.73 [0.45, 1.00]	0.52***	0.14	< 0.001
	Category 3 (interesting)	a3	0.64 [0.33, 0.96]	0.35***	0.16	< 0.001
	Category 4^*a*^ (unconcerned)			–	–	−
Geriatric Depression Scale	Meaning reflectivity					
	Category 1 (talked about)	c1	–0.22 [–0.76, 0.33]	–0.10	0.28	0.44
	Category 2 (thought about)	c2	–0.49 [–0.96, –0.02]	–0.25*	0.24	0.04
	Category 3 (interesting)	c3	–0.49 [–1.02, 0.03]	–0.20	0.27	0.07
	Category 4^*a*^ (unconcerned)			–	–	−
	Presence of Meaning	b	–0.27 [–0.43, –0.10]	–0.19**	0.09	0.002
	Indirect effects					
	a1b		–0.17 [–0.28, –0.05]	–0.07**	0.06	0.006
	a2b		–0.19 [–0.32, –0.07]	–0.10**	0.06	0.002
	a3b		–0.17 [–0.30, –0.05]	–0.07**	0.06	0.006
	Total effects					
	a1b + c1		–0.38 [–0.92, 0.16]	–0.17	0.28	0.17
	a2b + c2		–0.68 [–1.16, –0.20]	–0.35**	0.25	0.005
	a3b + c3		–0.66 [–1.20, –0.13	–0.26*	0.27	0.02

N = 282. Paths from the covariates (gender, age, education, civil state) not shown for clarity (see OSF for full model output: https://osf.io/rnzad). B, unstandardized coefficients; SE, standard errors; CI, confidence interval. Category 1 = “Yes, I have talked about this with others,” Category 2 = “Yes, I have thought about it before,” Category 3 = “No, but I do find it interesting,” Category 4 = “No, that doesn’t concern me.” ^*a*^Reference category. ***p < 0.001; **p < 0.01; *p < 0.05.

## Discussion

In the present paper, we examined to what extent community-dwelling older adults in Belgium engage in meaning reflectivity and how this meaning reflectivity is related to their experience of meaning in life (both presence and search) and depressive symptoms. As expected, the majority of older adults had either thought (42.4%) or talked (23.2%) about meaning in their life before participating in the study. However, a substantial proportion of participants had not thought about meaning in their life before. About half of these participants (18% of the total sample) indicated that they did find it an interesting topic, while the other half (16.4% of the total sample) declared that they were unconcerned with the topic. When comparing these four groups, especially the latter group differed from the other groups on the outcomes examined: they experienced less presence of meaning in their life, where searching for it less, and reported more depressive symptoms. Looking back at previous research, the mean levels of presence of meaning and search for meaning for this group were also lower than was observed in a general sample of community dwelling older adults aged 70 or more (Van der Heyden et al., 2015). In contrast, their scores were more similar to the relatively lower levels that have been observed in a nursing home population (Dewitte et al., 2019). While it should be noted that the mean score for depressive symptoms for the overall sample was low, the mean score for the unconcerned group also leaned more toward scores that have been observed in more challenged populations such as older adults recovering from stroke (Buijck et al., 2014) or older adults living in nursing homes (Dezutter et al., 2020).

Meaning reflectivity categories also statistically predicted higher presence of meaning and search for meaning adjusted for covariates, but did not predict depressive symptoms above the predictive effect of presence of meaning. An exploratory mediation model suggested that meaning reflectivity may be indirectly associated with depressive symptoms through presence of meaning.

These results are in line with a limited number of previous findings in the general population which found that reflecting on meaning or other existential topics is related to higher meaning in life and/or better psychological functioning (Allan and Shearer, 2012; Hill et al., 2019). With the current study, we thus extend this finding to a population of older adults specifically. However, previous work suggests that reflecting on topics like meaning in life may not always be equally beneficial. More specifically, adults without a guiding religious framework or adults living in a highly globalized society may experience difficulties in finding satisfying answers to questions of meaning when reflecting on it, which may be accompanied by a decrease rather than increase in psychological well-being (Joshanloo and Weijers, 2014). The context of the current study (Belgium) can be regarded as a globalized and secular country, but older adults often remain more religious. Although we could not investigate the role of religion in the current study, this may be one possible explanation for the positive association found between meaning reflectivity and adaptive outcomes (presence of meaning and less depressive symptoms). For older adults nearing the end of their life, existential topics can become more salient. It may be easier for those who can rely on a firm religious belief system to find a confirmation of life’s meaning and a heightened sense of well-being when going through an existential reflection process. However, future studies specifically assessing religious affiliation and activities in older adults are needed to confirm this hypothesis.

The statistical models of the current studies were based on the theoretical framework that meaning reflectivity proceeds and predicts the experience of meaning in life and, in turn, psychological health (Allan and Shearer, 2012; Hill et al., 2019). However, given the cross-sectional nature of the study, we were unable to test the temporal direction of our hypothesized effects. A concept related to meaning reflectivity has been forwarded by Hooker et al. (2018). They propose the relevance of *meaning salience—*or “the extent to which meaning stands out or is conspicuous to individuals” (p. 16)*—*for explaining the positive relation between meaning in life and health outcomes. However, in this view, meaning salience follows the experience of meaning rather than being a preceding process. Longitudinal and experimental studies will be needed to disentangle the temporal relationship between meaning in life on the one hand and meaning reflectivity and related concepts such as meaning salience on the other hand, although it seems reasonable to suspect that experiencing meaning in life and reflecting on this experience are likely to be in a reciprocal interaction.

Although the current findings should be interpreted with caution given a number of limitations (see below), they provide an important first indication for the relevance of the concept of meaning reflectivity for the psychological functioning of older adults. In doing so, the current study sheds first empirical light on a potential *process* of daily meaning construction in later life. These findings and future work in this area can provide useful information for the further development of effective meaning interventions. For example, one common strategy to support older adults who are struggling to find meaning in their life is the use of life-review interventions (Westerhof et al., 2010). Such interventions provide a structured setting in which older adults are encouraged to review their life story, integrate negative and positive memories, and relate these experiences to the present circumstances and future aspirations. Including meaning reflectivity as an explicit component in these interventions may hold promise in leveraging its positive effects. In fact, some studies that examined the potential of a meaning-focused life-review approach (called “spiritual reminiscence”) have shown promising results in older adults with dementia (MacKinlay and Trevitt, 2010; Wu and Koo, 2016; Ching-Teng et al., 2020). Similarly, meaning reflectivity could be incorporated more explicitly in existing and effective programs such as gratitude diary interventions (Killen and Macaskill, 2015) or interventions focusing on gerotranscendence (Wang et al., 2011).

Of course, many other processes besides meaning reflectivity are likely involved. In line with prominent dual process theories of thinking in fields like learning and decision making (e.g., Evans, 2010; Kahneman, 2011), we may expect that both more effortful processes like meaning reflectivity and more spontaneous processes are involved. This idea has for example also been proposed by Ward and King (2017), who reviewed existing evidence suggesting that both reflective and intuitive information processing styles are involved in making sense of our experiences. The current findings can be used to further build this underdeveloped line of research.

### Limitations

This research has some important limitations to take into account. First, the use of a non-validated measure to divide participants into categories of meaning reflectivity is an important shortcoming. Our findings demonstrate the relevance of meaning reflectivity for the future study of meaning in life, but future studies would benefit from a validated scale. After the data for the current study was collected, Hill et al. (2019) proposed a dimensional scale to measure meaning reflectivity. Further work is needed to clarify whether this concept is best operationalized as a categorical or dimensional variable.

Second, the Presence of Meaning subscale assesses participants’ overall experience of meaning in life. However, following the growing consensus on the tripartite structure of meaning in life, more recent scales have been developed, which tap into the three components of coherence, purpose, and significance separately. It is an interesting question for future studies whether or not meaning reflectivity relates differently to the three subcomponents of meaning in life. It could, for example, be hypothesized that this reflective process is more important in constructing the cognitive component of coherence.

Third, the cross-sectional nature of the study precludes any conclusions about the temporal or causal relation between the variables. Especially the explorative mediation model should be interpreted with strong caution, as cross-sectional mediation analyses often produce biased estimates of presumed longitudinal effects, both for complete and partial mediation effects (Maxwell and Cole, 2007; Maxwell et al., 2011). Moreover, a statistical mediation model relies on strong assumptions such as the absence of both confounding and reverse causality—assumptions which are not unlikely to be violated in the present study (Rohrer et al., 2021). As mentioned above, we acknowledge the possibility of an opposite or bidirectional effect between meaning reflectivity and meaning in life. Furthermore, we can think of several unmeasured confounding variables that are possibly related to both meaning reflectivity and meaning in life, such as cognitive style, personality traits (e.g., extraversion and openness), and spirituality or religiosity (Schnell and Becker, 2006; Steger et al., 2008a). Because we are eventually interested in causal inferences, we included the mediation model based on our theoretical hypotheses, but we emphasize the need for further longitudinal or experimental work in this area.

Fourth, participants of this study were a convenience sample of older adults. This data collection approach involves an important self-selection bias on the level of the participants; older adults who decide to participate in a study like this may differ from participants who decline to participate in important ways. For example, it is not unlikely that the most vulnerable older adults, like those with more severe physical disability, depressive symptoms, or smaller interpersonal networks are less likely to participate. Looking at the mean score of depressive symptoms in our study, we indeed see a low value of less than one on a scale from zero to eight (although with considerable variance). This limits the generalizability of the current findings to the general population of older adults living at home. The current findings can also not be readily generalized to other specific old age populations, such as those living in nursing homes.

## Conclusion

The extent to which older adults think or talk about meaning in their lives (i.e., meaning reflectivity) is a relevant concept to consider when studying meaning in later life. We showed that most older adults engage in some form of meaning reflectivity (either thinking or talking about it) or are interested in the topic of meaning. However, a substantial proportion (about one in six) of older adults report to be unconcerned with the topic of meaning life. Importantly, belonging to this category was predictive of experiencing lower meaning in life and a higher rates of depressive symptoms. Meaning reflectivity may thus be an important process involved in the experience of meaning and may therefore be an interesting mechanism to focus on when developing potential interventions for older adults experiencing a lack of meaning in life.

## Data Availability Statement

The raw data supporting the conclusions of this article will be made available by the authors, without undue reservation.

## Ethics Statement

The study involving human participants was reviewed and approved by the Social and Societal Ethics Committee (SMEC) of KU Leuven (G- 2017 03 803). The participants provided their written informed consent to participate in this study.

## Author Contributions

This study was designed by JD and LD, in discussion with other lab members of JD. JD supervised the data collection and provided critical feedback for manuscript revision. LD analyzed the data and wrote the first draft of the manuscript. Both authors contributed to the article and approved the submitted version.

## Conflict of Interest

The authors declare that the research was conducted in the absence of any commercial or financial relationships that could be construed as a potential conflict of interest.

## Publisher’s Note

All claims expressed in this article are solely those of the authors and do not necessarily represent those of their affiliated organizations, or those of the publisher, the editors and the reviewers. Any product that may be evaluated in this article, or claim that may be made by its manufacturer, is not guaranteed or endorsed by the publisher.

## Footnotes

1
osf.io/sa4d5/

2
https://osf.io/rnzad

## References

[B1] Allan B. A. Shearer C. B. (2012). The scale for existential thinking. *Int. J. Transpers. Stud.* 31 21–37. 10.24972/ijts.2012.31.1.21

[B2] Bar-Tur L. Savaya R. Prager E. (2001). Sources of meaning in life for young and old Israeli Jews and Arabs. *J. Aging Stud.* 15 253–269. 10.1016/S0890-4065(01)00022-6

[B3] Bengtson V. L. Silverstein M. Putney N. M. Harris S. C. (2015). Does religiousness increase with age? Age changes and generational differences over 35 years. *J. Sci. Study Religion* 54 363–379. 10.1111/jssr.12183

[B4] Boyle P. A. Buchman A. S. Barnes L. L. Bennett D. A. (2010). Effect of a purpose in life on risk of incident Alzheimer disease and mild cognitive impairment in community-dwelling older persons. *Arch. Gen. Psychiatry* 67:304. 10.1001/archgenpsychiatry.2009.208 20194831 PMC2897172

[B5] Boyraz G. Efstathiou N. (2011). Self-focused attention, meaning, and posttraumatic growth: the mediating role of positive and negative affect for bereaved women. *J. Loss Trauma* 16 13–32. 10.1080/15325024.2010.507658

[B6] Buijck B. I. Zuidema S. U. Spruit-van Eijk M. Bor H. Gerritsen D. L. Koopmans R. T. (2014). Determinants of geriatric patients’ quality of life after stroke rehabilitation. *Aging Ment. Health* 18 980–985. 10.1080/13607863.2014.899969 24679003

[B7] Ching-Teng Y. Ya-Ping Y. Chia-Ju L. Hsiu-Yueh L. (2020). Effect of group reminiscence therapy on depression and perceived meaning of life of veterans diagnosed with dementia at veteran homes. *Soc. Work Health Care* 59 75–90. 10.1080/00981389.2019.1710320 31944912

[B8] Cohen G. D. (2005). *The Mature Mind: The Positive Power of the Aging Brain.* New York, NY: Basic Books (AZ).

[B9] Davis C. G. Nolen-Hoeksema S. Larson J. (1998). Making sense of loss and benefiting from the experience: two construals of meaning. *J. Pers. Soc. Psychol.* 75 561–574. 10.1037/0022-3514.75.2.561 9731325

[B10] Debats D. L. (1999). Sources of meaning an investigation of significant commitments in life [Article; Proceedings Paper]. *J. Hum. Psychol.* 39 30–57. 10.1177/0022167899394003

[B11] Delle Fave A. Brdar I. Wissing M. P. Vella-Brodrick D. A. (2013). Sources and motives for personal meaning in adulthood. *J. Posit. Psychol.* 8 517–529. 10.1080/17439760.2013.830761

[B12] Dewitte L. Vandenbulcke M. Schellekens T. Dezutter J. (2019). Sources of well-being for older adults with and without dementia in residential care: relations to presence of meaning and life satisfaction. *Aging Ment. Health* 25 1–9. 10.1080/13607863.2019.1691144 31729244

[B13] Dezutter J. Toussaint L. Dewitte L. (2020). Finding a balance between integrity and despair: a challenging task for older adults in residential care. *J. Adult Dev.* 27 147–156. 10.1007/s10804-019-09332-1

[B14] Evans J. S. B. (2010). Intuition and reasoning: a dual-process perspective. *Psychol. Inq.* 21 313–326. 10.1080/1047840X.2010.521057

[B15] Fegg M. J. Kramer M. Bausewein C. Borasio G. D. (2007). Meaning in life in the federal Republic of Germany: results of a representative survey with the schedule for meaning in life evaluation (SMiLE) [Article]. *Health Qual. Life Outcomes* 5:97. 10.1186/1477-7525-5-59 18034898 PMC2206010

[B16] Frankl V. E. (1968). *Man’s Search for Meaning: An Introduction to Logotherapy.* London: Hodder and Stoughton.

[B17] Gardner W. Mulvey E. P. Shaw E. C. (1995). Regression analyses of counts and rates: poisson, overdispersed poisson, and negative binomial models. *Psychol. Bull.* 118:392. 10.1037/0033-2909.118.3.392 7501743

[B18] George L. S. Park C. L. (2016). Meaning in life as comprehension, purpose, and mattering: toward integration and new research questions. *Rev. Gen. Psychol.* 20 205–220. 10.1037/gpr0000077

[B19] Glaesmer H. Riedel-Heller S. Braehler E. Spangenberg L. Luppa M. (2011). Age-and gender-specific prevalence and risk factors for depressive symptoms in the elderly: a population-based study. *Int. Psychogeriatr.* 23:1294. 10.1017/S1041610211000780 21729425

[B20] Graham J. W. (2009). Missing data analysis: making it work in the real world. *Annu. Rev. Psychol.* 60 549–576. 10.1146/annurev.psych.58.110405.085530 18652544

[B21] Heine S. J. Proulx T. Vohs K. (2006). The meaning maintenance model: on the coherence of social motivations. *Pers. Soc. Psychol. Rev.* 10 88–110. 10.1207/s15327957pspr1002_1 16768649

[B22] Heintzelman S. J. King L. A. (2014). (The feeling of) meaning-as-information. *Pers. Soc. Psychol. Rev.* 18 153–167. 10.1177/1088868313518487 24501092

[B23] Hill C. E. Kline K. V. Miller M. Marks E. Pinto-Coelho K. Zetzer H. (2019). Development of the meaning in life measure. *Couns. Psychol. Q.* 32 205–226. 10.1080/09515070.2018.1434483

[B24] Hill P. L. Weston S. J. (2019). Evaluating eight-year trajectories for sense of purpose in the health and retirement study. *Aging Ment. Health* 23 233–237. 10.1080/13607863.2017.1399344 29212348

[B25] Hooker S. A. Masters K. S. Park C. L. (2018). A meaningful life is a healthy life: a conceptual model linking meaning and meaning salience to health. *Rev. Gen. Psychol.* 22 11–24. 10.1037/gpr0000115

[B26] Hupkens S. Machielse A. Goumans M. Derkx P. (2018). Meaning in life of older persons: an integrative literature review. *Nurs. Ethics* 25 973–991. 10.1177/0969733016680122 30871429

[B27] Irving J. Davis S. Collier A. (2017). Aging with purpose: systematic search and review of literature pertaining to older adults and purpose. *Int. J. Aging Human Dev.* 85 403–437. 10.1177/0091415017702908 28391702

[B28] Jongenelis K. Gerritsen D. Pot A. Beekman A. Eisses A. Kluiter H. (2007). Construction and validation of a patient-and user-friendly nursing home version of the Geriatric depression scale. *Int. J. Geriatr. Psychiatry* 22 837–842. 10.1002/gps.1748 17199236

[B29] Joshanloo M. Weijers D. (2014). Does thinking about the meaning of life make you happy in a religious and globalised world? A 75-nation study. *J. Psychol. Afr.* 24 73–81. 10.1080/14330237.2014.904093

[B30] Kahneman D. (2011). *Thinking, Fast and Slow.* New York, NY: Farrar, Straus and Giroux.

[B31] Killen A. Macaskill A. (2015). Using a gratitude intervention to enhance well-being in older adults. *J. Happiness Stud.* 16 947–964. 10.1007/s10902-014-9542-3

[B32] Krause N. (2007). Longitudinal study of social support and meaning in life. *Psychol. Aging* 22 456–469. 10.1037/0882-7974.22.3.456 17874947

[B33] Kretschmer M. Storm L. (2018). The relationships of the five existential concerns with depression and existential thinking. *Int. J. Existent. Posit. Psychol.* 7:20.

[B34] MacKinlay E. Trevitt C. (2010). Living in aged care: using spiritual reminiscence to enhance meaning in life for those with dementia. *Int. J. Ment. Health Nurs.* 19 394–401. 10.1111/j.1447-0349.2010.00684.x 21054725

[B35] Martela F. Steger M. F. (2016). The three meanings of meaning in life: distinguishing coherence, purpose, and significance. *J. Posit. Psychol.* 11 531–545. 10.1080/17439760.2015.1137623

[B36] Maxwell S. E. Cole D. A. (2007). Bias in cross-sectional analyses of longitudinal mediation. *Psychol. Methods* 12:23. 10.1037/1082-989X.12.1.23 17402810

[B37] Maxwell S. E. Cole D. A. Mitchell M. A. (2011). Bias in cross-sectional analyses of longitudinal mediation: partial and complete mediation under an autoregressive model. *Multivariate Behav. Res.* 46 816–841. 10.1080/00273171.2011.606716 26736047

[B38] Minicuci N. Maggi S. Pavan M. Enzi G. Crepaldi G. (2002). Prevalence rate and correlates of depressive symptoms in older individuals: the Veneto Study. *J. Gerontol. Ser. A Biol. Sci. Med. Sci.* 57 M155–M161. 10.1097/JGP.0b013e3181e70d09 11867651

[B39] Newman D. B. Nezlek J. B. (2019). Private self-consciousness in daily life: relationships between rumination and reflection and well-being, and meaning in daily life. *Pers. Individ. Differ.* 136 184–189. 10.1016/j.paid.2017.06.039

[B40] Park C. L. (2010). Making sense of the meaning literature: an integrative review of meaning making and its effects on adjustment to stressful life events. *Psychol. Bull.* 136 257–301. 10.1037/a0018301 20192563

[B41] Park C. L. George L. S. (2018). Lab-and field-based approaches to meaning threats and restoration: convergences and divergences. *Rev. Gen. Psychol.* 22:73. 10.1037/gpr0000118

[B42] Park N. Park M. Peterson C. (2010). When is the search for meaning related to life satisfaction? *Appl. Psychol. Health Well Being* 2 1–13. 10.1111/j.1758-0854.2009.01024.x

[B43] Pinquart M. (2002). Creating and maintaining purpose in life in old age: a meta-analysis. *Ageing Int.* 27 90–114. 10.1007/s12126-002-1004-2

[B44] Reker G. T. Wong P. T. P. (2012). “Personal meaning in life and psychosocial adaptation in the later years,” in *The Human Quest for Meaning: Theories, Research, and Applications*, 2nd Edn, ed. Wong P. T. P. (London: Routledge), 433–456.

[B45] Rohrer J. M. Hünermund P. Arslan R. C. Elson M. (2021). That’sa lot to PROCESS! Pitfalls of popular path models. *PsyArXiv* [Preprint]. 10.31234/osf.io/paeb7

[B46] Rosseel Y. (2012). Lavaan: an R package for structural equation modeling and more. Version 0.5–12 (BETA). *J. Stat. Softw.* 48 1–36. 10.18637/jss.v048.i02

[B47] Schnell T. (2009). The sources of meaning and meaning in life questionnaire (SoMe): relations to demographics and well-being [Article]. *J. Posit. Psychol.* 4 483–499. 10.1080/17439760903271074

[B48] Schnell T. Becker P. (2006). Personality and meaning in life. *Pers. Individ. Differ.* 41 117–129. 10.1016/j.paid.2005.11.030

[B49] Steger M. F. Frazier P. Oishi S. Kaler M. (2006). The meaning in life questionnaire: assessing the presence of and search for meaning in life. *J. Couns. Psychol.* 53:80. 10.1037/0022-0167.53.1.80

[B50] Steger M. F. Kashdan T. B. Sullivan B. A. Lorentz D. (2008a). Understanding the search for meaning in life: personality, cognitive style, and the dynamic between seeking and experiencing meaning. *J. Pers.* 76 199–228. 10.1111/j.1467-6494.2007.00484.x 18331281

[B51] Steger M. F. Kawabata Y. Shimai S. Otake K. (2008b). The meaningful life in Japan and the United States: levels and correlates of meaning in life. *J. Res. Pers.* 42 660–678. 10.1016/j.jrp.2007.09.003

[B52] Steger M. F. Oishi S. Kashdan T. B. (2009). Meaning in life across the life span: levels and correlates of meaning in life from emerging adulthood to older adulthood. *J. Posit. Psychol.* 4 43–52. 10.1080/17439760802303127

[B53] Steger M. F. Samman E. (2012). Assessing meaning in life on an international scale: psychometric evidence for the meaning in life questionnaire-short form among chilean households. *Int. J. Wellbeing* 2 182–195. 10.5502/ijw.v2i.i3.2 32285354

[B54] Steptoe A. Fancourt D. (2019). Leading a meaningful life at older ages and its relationship with social engagement, prosperity, health, biology, and time use. *Proc. Natl. Acad. Sci. U.S.A.* 116 1207–1212. 10.1073/pnas.1814723116 30617082 PMC6347683

[B55] Sterne J. A. White I. R. Carlin J. B. Spratt M. Royston P. Kenward M. G. (2009). Multiple imputation for missing data in epidemiological and clinical research: potential and pitfalls. *BMJ* 338 b2393. 10.1136/bmj.b2393 19564179 PMC2714692

[B56] Tornstam L. (1989). Gero-transcendence: a reformulation of the disengagement theory. *Aging Clin. Exp. Res.* 1 55–63. 10.1007/BF03323876 2488301

[B57] Tornstam L. (1997). Gerotranscendence: the contemplative dimension of aging. *J. Aging Stud.* 11 143–154. 10.1016/S0890-4065(97)90018-9

[B58] Van der Heyden K. Dezutter J. Beyers W. (2015). Meaning in life and depressive symptoms: a person-oriented approach in residential and community-dwelling older adults. *Aging Mental Health* 19 1063–1070. 10.1080/13607863.2014.995589 25555243

[B59] van’t Veer-Tazelaar P. J. N. van Marwijk H. W. Jansen A. P. D. Rijmen F. Kostense P. J. van Oppen P. (2008). Depression in old age (75+), the PIKO study. *J. Affect. Disord.* 106 295–299. 10.1016/j.jad.2007.07.004 17720253

[B60] Volkert J. Härter M. Dehoust M. C. Ausín B. Canuto A. Da Ronch C. (2019). The role of meaning in life in community-dwelling older adults with depression and relationship to other risk factors. *Aging Mental Health* 23 100–106. 10.1080/13607863.2017.1396576 29115865

[B61] Wang J.-J. Lin Y.-H. Hsieh L.-Y. (2011). Effects of gerotranscendence support group on gerotranscendence perspective, depression, and life satisfaction of institutionalized elders. *Aging Mental Health* 15 580–586. 10.1080/13607863.2010.543663 21815850

[B62] Ward S. King L. (2017). “Making sense: meaning in life in a cognitive context,” in *The Happy Mind: Cognitive Contributions to Well-Being*, eds Robinson M. D. Eid M. (Cham: Springer International Publishing), 409–425. 10.1007/978-3-319-58763-9_22

[B63] Westerhof G. J. Bohlmeijer E. Webster J. D. (2010). Reminiscence and mental health: a review of recent progress in theory, research and interventions. *Ageing Soc.* 30:697. 10.1017/S0144686X09990328

[B64] Windsor T. D. Curtis R. G. Luszcz M. A. (2015). Sense of purpose as a psychological resource for aging well. *Dev. Psychol.* 51:975. 10.1037/dev0000023 26010384

[B65] Wong P. T. P. (2016). “Meaning-seeking, self-transcendence, and well-being,” in *Logotherapy and Existential Analysis: Proceedings of the Viktor Frankl Institute Vienna*, Vol. 1 ed. Batthyany A. (New York, NY: Springer International Publishing), 311–321. 10.1007/978-3-319-29424-7_27

[B66] Wu L. F. Koo M. (2016). Randomized controlled trial of a six-week spiritual reminiscence intervention on hope, life satisfaction, and spiritual well-being in elderly with mild and moderate dementia. *Int. J. Geriatr. Psychiatry* 31 120–127. 10.1002/gps.4300 25965388

[B67] Yount W. R. (2008). Transcendence and aging: the secular insights of Erikson and Maslow. *J. Relig. Spiritual. Aging* 21 73–87. 10.1080/15528030802265361

